# Canine Distemper Virus Infection in the Free-Living Wild Canines, the Red Fox (*Vulpes vulpes*) and Jackal (*Canis aureus moreoticus*), in Croatia

**DOI:** 10.3390/pathogens12060833

**Published:** 2023-06-15

**Authors:** Jelena Prpić, Ivana Lojkić, Tomislav Keros, Nina Krešić, Lorena Jemeršić

**Affiliations:** Croatian Veterinary Institute, Savska cesta 143, 10000 Zagreb, Croatia

**Keywords:** canine distemper virus, real-time, RT-PCR, phylogenetic analysis, wild canids

## Abstract

The canine distemper virus (CDV), a paramyxovirus that is closely related to the human measles virus and rinderpest virus of cattle, is a highly contagious viral disease in dogs and wild carnivores worldwide. CDV represents a serious threat to domestic and wild animals, especially to the conservation of endangered wild carnivores. Our study aims to investigate the occurrence of CDV in free-living wild canines in Croatia. For this purpose, 176 red foxes and 24 jackal brain samples collected in the frame of the active surveillance of rabies during winter 2021/2022 were tested. This study provided the first comprehensive overview of the prevalence and spatial distribution of CDV in the wildlife of Croatia, including the molecular phylogenetic analysis of the H gene sequence of field CDV strains circulating in red fox and jackal populations of Croatia. The molecular characterization of hemagglutinin gene genomic regions confirmed the phylogenetic clustering of obtained sequences into the Europa 1 genotype. The obtained CDV red fox sequences were mutually very similar (97.60%). This study indicates the high genetic similarity of Croatian CDV red fox sequences and CDV red fox sequences from Italy and Germany, badger sequences from Germany, polecat sequences from Hungary, and dog sequences from Hungary and Germany.

## 1. Introduction

Due to human activities and constant environmental changes [1,2], a variety of emerging and re-emerging viral diseases are shared between wildlife and domestic animals [1,2], posing a major threat to the global economy and health. This is the case with *Canine morbillivirus* (previously called distemper virus, CDV), one of the members of the genus *Morbillivirus*, family *Paramyxoviridae* [3]. CDV has spread worldwide and is associated with highly contagious infections in carnivores, especially canids [4], which represents a serious threat to both wild and domestic animals.

CDV is an enveloped virus with a non-segmented single-stranded negative-sense RNA genome of approximately 15,690 bp, which encodes for six proteins: nucleocapsid (N), phospho- (P), matrix (M), fusion (F), hemagglutinin (H) and polymerase (L) proteins. One of the most variable genes is the hemagglutinin (H) gene [5] which is a widely used target for molecular epidemiological studies [6,7]. Based on the phylogenetic analysis of H gene sequences, at least 20 genotypes of CDV have been identified, including the recently discovered Caspian and Asia 4–6 genotypes [8,9,10,11,12].

Previous studies have reported CDV circulation in wildlife species (Euroasian badger, *Meles meles*; stone marten, *Marten foina* and red fox, *Vulpes vulpes*) in Germany, Italy, Denmark, and Switzerland [13,14,15,16,17,18,19,20,21]. Additionally, there is a report of sporadic cases of CDV in wild mustelids in the Czech Republic [22]. Previous serological or viral nucleic acid surveys have indicated CDV circulation in the red fox, Iberian wolf (*Canis lupus*), and Iberian lynx (*Lynx pardinus*) populations in Spain and Portugal [23,24,25,26,27].

Therefore, there is only a limited number of studies regarding CDV prevalence in wildlife available, and these studies are mostly targeted and based on investigations in dead animals or those with neurological symptoms. The aim of our study was to investigate the prevalence of CDV in Croatian wild canine species, red foxes, and jackals (*Canis aureus moreoticus*), collected in the frame of the active surveillance of rabies and, therefore, healthy hunted individuals. Additionally, the molecular characterization of H gene sequences was used to determine currently circulating CDV lineages in Croatia.

## 2. Materials and Methods

### 2.1. Sample Collection and Preparation

**Figure 1:**
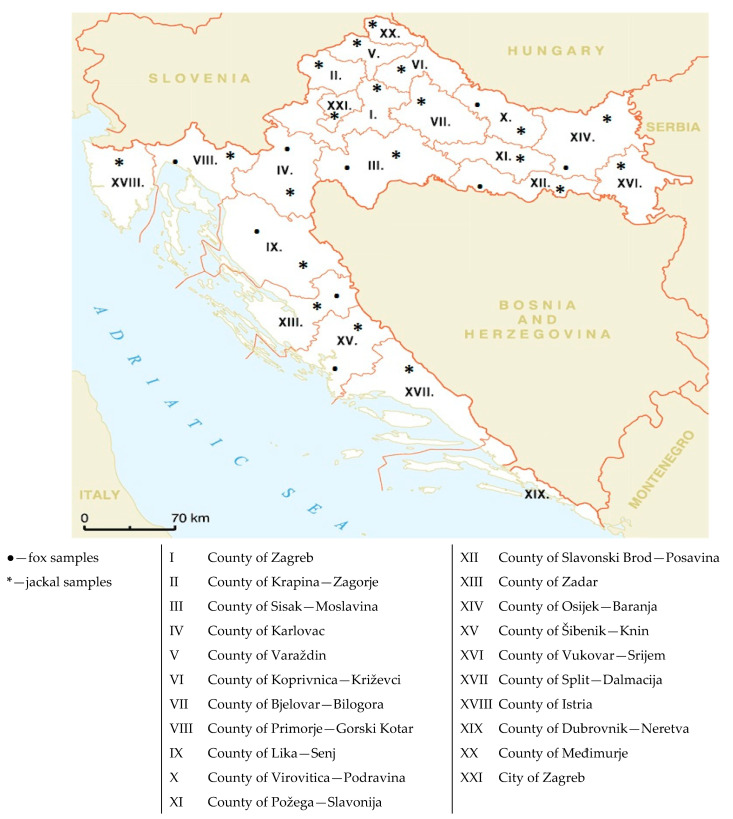
Map of Croatia indicating the regions where the red fox and jackal samples were collected.

The brain samples of red foxes and European jackals were collected during November and December 2021 according to an ongoing national rabies annual monitoring program prescribed by the Croatian Ministry of Agriculture, Veterinary and Food Safety Directorate. Samples were randomly chosen, taking into account the sample quality and geographical origin (Figure 1). Brain samples were collected immediately after the animal’s death and were taken from fox/jackal carcasses by trained pathology technicians at the Croatian Veterinary Institute Pathology laboratory. These samples were placed in polypropylene containers (security screw cap containers, 120 mL, DeltaLab, Rubi Barcelona, Spain) and stored at −80 °C until use. Brain samples were initially tested with a direct fluorescent antibody test (DFA) for the presence of the rabies virus [28]. In addition to the samples from wildlife, a canine vaccine containing a live attenuated CDV strain Onderstepoort (Nobivac DHPPi, MSD Animal Health, Rahway, NJ, USA) was used for PCR and sequencing control.

In total, 176 red foxes and 24 jackals were tested for CDV RNA presence (Table 1).

For RNA extractions, brain samples were initially homogenized in Dulbecco’s Modified Eagles Medium (DMEM) to gain 10% (*w*/*v*) brain suspensions. These brain suspensions were centrifuged for 10 min at 220× *g* and then used as the starting material for RNA extraction. To monitor RNA extraction and the appearance of potential PCR inhibitors, the samples were also screened for the presence of the mammalian beta-actin gene [29].

### 2.2. Detection of CDV RNA

Viral RNA was extracted from 200 μL of the supernatant of centrifuged suspensions using a MagMAX Core Kit (Thermo Fisher Scientific, Waltham, MA, USA) on a KingFisher™ Duo Prime System (Thermo Fisher Scientific, Waltham, MA, USA) according to the manufacturer’s instructions. RNA extracts were stored at −80 °C until use. For the detection of the conserved 83-bp fragment within the N protein region [30], a one-step real-time RT-PCR protocol was carried out. In brief, the amplification was carried out with a commercially available kit (AgPath-ID™ One-Step RT-PCR Kit, Thermo Fisher Scientific, Waltham, MA, USA), primers CDV-F (5′-AGCTAGTTTCATCTTAACTATCAAATT-3′) and CDV-R (5′-TTAACTCTCCAGAAAACTCATGC-3′), and the probe FAM CDV-Pb (5′-6-FAM-ACCCAAGAGCCGGATACATAGTTTCAATGC-TAMRA3′) according to the producer’s instructions. The amplification was carried out in a Rotorgene Q (Qiagen, Hilden, Germany) according to an established protocol [31]. The samples containing CDV RNA, which were detectable at a cycle threshold value lower than 37, were re-tested by an RT-PCR protocol [21] for the detection of a variable 484 bp fragment within the H gene region. Conventional RT-PCR was performed using the Superscript III One-Step RT-PCR system with a Platinum Taq DNA Polymerase (Thermo Fisher Scientific, Waltham, MA, USA) according to an established protocol (reverse transcription for 30 min at 50 °C, RT inactivation/PCR activation for 2 min at 94 °C, 35 cycles of 45 s denaturation at 94 °C, 45 s annealing at 48 °C and 60 s elongation at 68 °C). In each test, RNA was isolated from the Nobivac DHPPi vaccine (MSD Animal Health, Rahway, NJ, USA), marked as Vaccine/D1 on a phylogenetic tree, and used as a positive control. A possible nucleic acid contamination was monitored using aliquots of ultrapure water.

### 2.3. Sequence and Phylogenetic Analysis

PCR products were purified with ExoSAP-IT™ PCR Product Cleanup Reagent (Thermo Fisher Scientific, Waltham, MA, USA) and sequenced in both directions (Macrogen Europe, B.V., Amsterdam, Netherlands). The 484 base pair sequences of Vaccine/D1, Croatia/D7/KK, and Croatia/D23/SM were submitted to GenBank (accession no. OQ857156-OQ857158). Phylogenetic grouping and clustering were based on a comparison with sequences retrieved from GenBank using the algorithm BLAST (http://www.ncbi.nlm.nih.gov). These sequences were aligned and compared using the ClustalX 2.1 program (European Bioinformatics Institute, Hinxton, Cambridgeshire, UK) and analyzed using MEGA11 [32], based on published recommendations [21]; by contrast, the trees were generated using the neighbor-joining method by applying the Kimura 2-parameter evolutionary model as the best-fit model estimated in MEGA11.

## 3. Results

### 3.1. Detection of CDV Specific RNA Fragments in Brain Samples

The screening assay targeting the conserved 83-bp fragment within the N protein coding region revealed four positive fox samples from Bjelovar-Bilogora, Koprivnica-Križevci, Sisak-Moslavina, and Vukovar-Srijem counties. The Ct values ranged from 28.53 to 38.18. Positive CDV RNA was not detected in the analyzed jackal samples (Table 2). Beta-actin was detected in all the extracted RNAs, indicating the absence of PCR inhibitors and the presence of the host material in the tested samples.

### 3.2. The Partial Sequencing of H Gene Region

**Figure 2:**
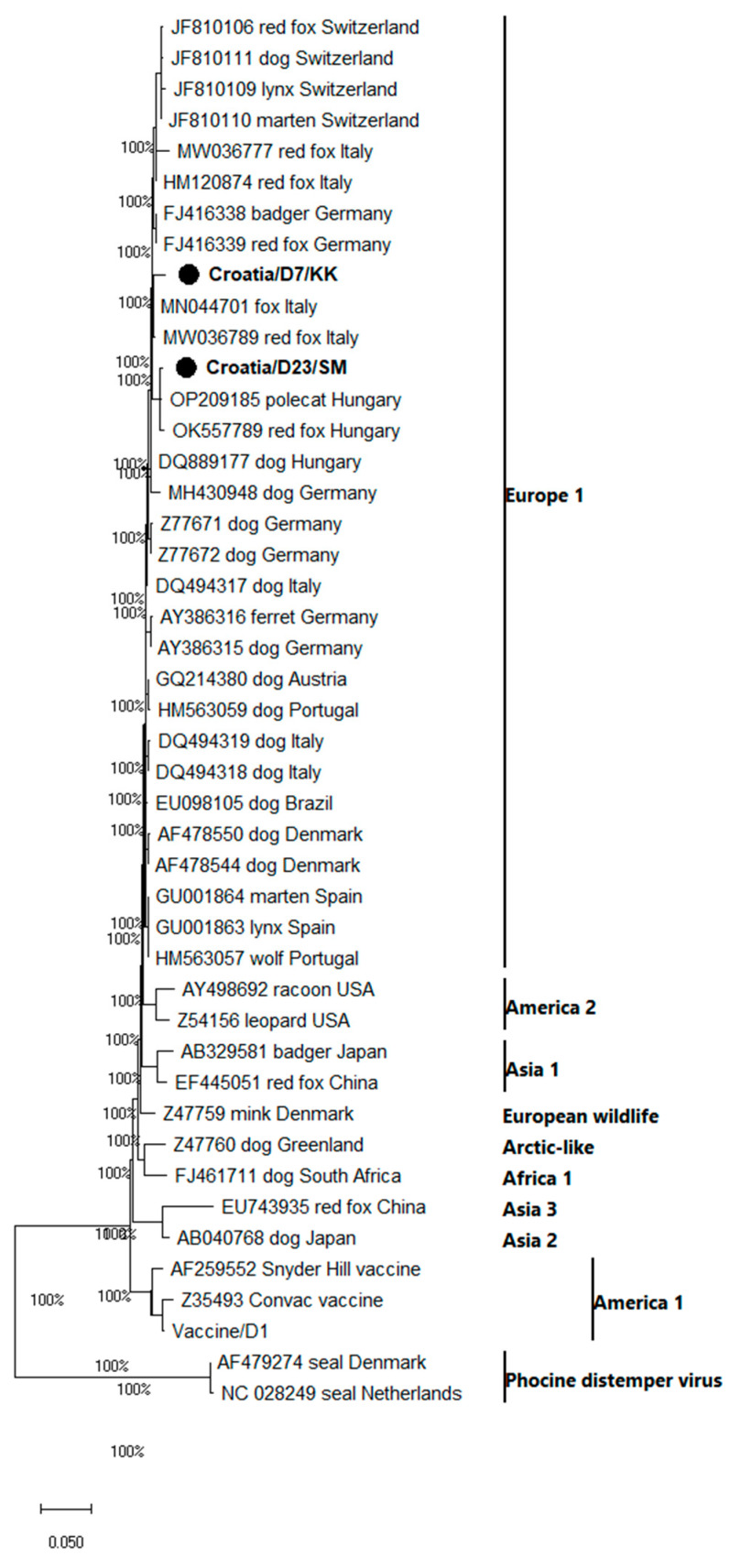
Phylogenetic analysis of partial H-gene sequences of CDV (375 nt). The tree was obtained by the neighbor-joining method using a Kimura-2 Parameter evolutionary model, the program MEGA11 and bootstrap values calculated from 1000 trees. The bar represents 0.05 nucleotide substitutions per site. The CDV sequences obtained in this study are marked with black dots, and geographic groups [21] are reported on the right side of the tree.

Among the four real-time RT-PCR positive samples, only two samples had Ct threshold values lower than 37, including the samples from Koprivnica-Križevci and Sisak-Moslavina counties (Croatia/D7/KK and Croatia/D23/SM), and these were furthermore amplified by a conventional RT-PCR (Table 2). The obtained partial H gene sequences were clustered within the CDV genetic group Europe 1 (Figure 2).

By comparison, a 97.60% nucleotide identity was found among Croatian CDV sequences. The partial H sequence from sample Croatia/D7/KK was most similar (98.67%) to a sequence from Italy (MN044701) isolated from a red fox, followed by sequences (98.4%) isolated from another red fox in Italy (MW036789), a red fox from Germany (FJ416339) and a badger from Germany (FJ416339). The comparison of Croatian/D23/SM sequences with reference database sequences showed the highest genetic similarities with a strain isolated from a Hungarian polecat (99.20%; OP209185), a Hungarian red fox (98.93%; OK557789), a Hungarian dog (98.67%; DQ889177) and a German dog (97.60%; MH430948).

## 4. Discussion

Canine distemper (CD) is an old viral disease that was first described by Antonio de Ulloa y de la Torre-Giral in 1746 as a disease affecting dogs in the Quito region and other parts of South America. Soon afterward, it was reported in Europe. CDV was recorded in Spain in 1760, and by 1764 and 1770, it had reached Great Britain and Italy, respectively [33]. Since the first report of this disease, CD has remained one of the most lethal canine diseases worldwide. Although CDV was originally thought to be restricted to canine host species, nowadays, it is regarded as a multi-host and globally distributed pathogen [34,35,36,37]. Its recent emergence into non-human primate species is a relevant cause for concern [38,39]. The integrated monitoring and investigation of the nature of the CD causative agent and its transmission pathways are essential tools for the early detection of disease outbreaks and, thus, for designing and optimizing effective disease prevention strategies [40]. CD surveillance in Europe is poorly documented. Available studies reported CDV circulation in Central Europe [13,14,41,42,43], which is persistent in Austria, Slovenia, and Croatia. During 2006–2010, a distemper epidemic spread in wild carnivores occurred throughout Northern Italy [44].

There are limited studies available on CDV distribution in red fox populations worldwide. In 2006, a CDV epidemic was reported in the red fox population in northern and northern Italy [15,44]. CDV was confirmed in red foxes with a prevalence of 10.7%, while other wild species appeared to have played a minor role in the epidemics [15]. Although available data on the role of jackals as potential reservoirs of zoonotic pathogens are scarce, the available studies show that jackals could play an important role in CDV dynamics [45]. It has been reported that black-backed jackals (*Canis mesomelas*) appear to have spread CDV between domestic dog populations during an outbreak along the Namibian coast [46]. It was presumed that CDV transmission in foxes primarily occurred through aerosolized respiratory excretions, as it was described in dogs [47]. Available reports have shown that the incubation period is variable, from 7 to 10 days up to 4 weeks, and CDV infection does not always have to be fatal for the canids [47]. The available field observations [47] detected a higher CDV prevalence (4%) in dead compared to living/apparently healthy foxes (2%). During the 1994 CDV epidemic in Serengeti lions, the main hypothesis was that CDV must have been introduced into the lion population from sympatric carnivores (jackals and hyenas) that may have been infected by domestic dogs [48]. Due to the rapid expansion of the jackal population, they may provide an epidemiological link between wildlife and domestic dogs, but their role in the transmission of multispecies carnivore pathogens remains unclear [45].

According to the data from the Ministry of Agriculture, the estimated number of red foxes in Croatia is 15,000, and the estimated jackal population size in Croatia is approximately 10,000. Over the last 15–20 years, the number of jackals and the localities they inhabit have rapidly increased. Herein, this is the first report of the CDV infection, prevalence, and characterization of CDV in healthy hunted red foxes in the frame of the oral vaccination campaign against rabies (ORV), which has been regularly implemented in Croatia since 2011 [49]. The detected prevalence of CDV in apparently healthy hunted red foxes from the Croatian territory is 2.27% which is lower than the prevalence previously detected in hunted foxes (4.83%) and before the implementation of ORV in Croatia [50] and dead foxes/foxes with neurological symptoms from the Italian territory [15]. Similar to other paramyxovirus infections, CDV infection primarily occurs during winter, which is the main reason why we collected samples during November and December. These obtained CDV sequences Croatia/D7/KK and Croatia/D23/SM were clustered within the genetic group Europe 1 and within the clade designated as Europe, and firstly described by [44]. Within the same cluster, isolates not only from red foxes in Italy, Germany, and Hungary (Figure 2) but also derived from badgers in Germany, a Siberian polecat (*Mustela eversmanii*) in Hungary and dogs in Hungary and Germany were found.

The high similarity of Croatian CDV red fox sequences and CDV red fox sequences from Italy, Germany, and Hungary suggests a possible transmission and spread through red fox dispersal. Moreover, a close genetic relationship between Croatian red fox strains and known badger and polecat CDV strains from the GenBank opens the question of a possible cross-species CDV transmission in Croatia. These species mostly share their prey with foxes, or in some cases, they are the fox’s prey or share the same habitat. There have been cases where foxes have lived in the distant tunnels of active badger setts, sometimes in a mutualistic relationship, or they inhabit the same tunnel systems [51]. Since there is a high genetic similarity between Croatian CDV red fox sequences and domestic dog sequences from Hungary and Germany, indicated by this study, the tendency of foxes to establish populations in suburban and urban areas should be considered a major risk factor for the transmission of such agents to domestic carnivores.

## 5. Conclusions

Further studies should be conducted to monitor the possible fluctuations in CDV incidence and prevalence with the consequent risk of transmission of CDV to domestic dogs and other wild mammals. Additionally, further studies regarding the prevalence of antibodies against CDV should be conducted in order to monitor the influence of age and season on the antibody prevalence to predict risk factors of CDV inter- and intraspecies transmission.

## Figures and Tables

**Table 1:** The number of tested red foxes and jackals per county.

County	Number of Red Fox Samples	Number of Jackal Samples
County of Zagreb	9	0
County of Krapina-Zagorje	7	0
County of Sisak-Moslavina	13	6
County of Karlovac	24	1
County of Varaždin	3	0
County of Koprivnica-Križevci	5	0
County of Bjelovar-Bilogora	14	0
County of Primorje-Gorski Kotar	9	1
County of Lika-Senj	30	4
County of Virovitica-Podravina	8	3
County of Požega-Slavonija	4	0
County of Slavonski Brod-Posavina	15	4
County of Zadar	2	1
County of Osijek-Baranja	7	3
County of Šibenik-Knin	8	1
County of Vukovar-Srijem	6	0
County of Split-Dalmacija	1	0
County of Istria	7	0
County of Dubrovnik-Neretva	0	0
County of Međimurje	2	0
City of Zagreb	2	0
Σ	176	24

**Table 2:** The results of CDV real-time RT-PCR and CDV RT-PCR detection in red fox and jackal brain samples.

	Σ	Real-Time RT-PCR	RT-PCR
Red fox samples	176	4 (2.27%)	2 (1.14%)
Jackal samples	24	0	0

## Data Availability

The sequences generated in this study are available in NCBI Genbank under the accession numbers OQ857156, OQ857157, OQ857158. The datasets used and/or analyzed within the frame of the present study can be provided by the corresponding author upon a justified request.
